# Development and application of a simple pharmacokinetic model that quantitatively describes the distribution and elimination of the commonly measured proteins

**DOI:** 10.5599/admet.1570

**Published:** 2023-01-26

**Authors:** David G. Levitt, Michael D. Levitt

**Affiliations:** 1 Department of Integrative Biology and Physiology, University of Minnesota, Minneapolis, MN 55455, USA; levit001@umn.edu; 2 Medicine Service, Veterans Affairs Medical Center, Minneapolis, MN. 55417, USA; michael.levitt@va.gov

**Keywords:** Protein, pharmacokinetics, alkaline phosphatase, alanine transaminase, aspartate aminotransferase, amylase, albumin, liver, pancreas

## Abstract

Increased plasma concentrations of a variety of cellular enzymes (alanine transaminase, aspartate aminotransferase, alkaline phosphatase, amylase, etc.) are commonly used as routine screening tests for a range of conditions. An increased concentration usually is assumed to result from an increased rate of delivery to the plasma. Factors such as decreased metabolism or excretion or altered extravascular distribution usually are ignored. As a prelude to a detailed analysis of all the factors producing altered plasma enzyme levels, we have reviewed the relevant literature describing the pharmacokinetics (PK) of 13 of the commonly measured plasma proteins and developed a PK model that provides a simple physiological description of all the data. Our model starts with the general 3-compartment, 6-parameter system previously developed for albumin and interprets the fluxes in terms of unidirectional sieved protein convectional volume flows from the plasma to the two tissue compartments and equal lymph flows returning to the plasma. This greatly constrains the model such that each protein is characterized by only two adjustable parameters (plasma clearance and sieving factor). In addition to accurately fitting the plasma kinetics, the model can accurately describe the tissue and lymph protein PK. For example, it can describe the thoracic duct lymph protein concentration following an intravenous infusion or the plasma concentration following a subcutaneous tissue injection. This simple model provides a satisfactory framework for the PK of 12 of the 13 proteins investigated. The glycoprotein intestinal alkaline phosphatase is the exception, requiring the addition of a liver recycling compartment involving the asialoglycoprotein receptor.

## Introduction

The standard approach to characterizing protein pharmacokinetics (PK) is via the classical 2-compartment (4 parameters) [1,2] or 3-compartment (6 parameters) [3,4] modeling. For most clinical purposes, 2 compartments are sufficient [1,2]. However, in order to accurately fit the details of the plasma concentration curve, usually 3 compartments are required [3,4]. Each protein is characterized by a six-parameter set determined by fitting the model to the plasma protein concentration following bolus injection.

The recent proliferation of therapeutic proteins, especially monoclonal antibodies, has led to an interest in developing physiologically based pharmacokinetic modeling (PBPK) of these proteins [5-8]. In this approach, each of the major organs is described in terms of their physiological parameters (blood flow, capillary permeability, lymph flow, interstitial volume, protein catabolism, etc.) and the PK is related to these parameters. The major theoretical advantage of this approach is that once these parameters are determined for, e.g., the human, they allow one to predict the human protein pharmacokinetics of a novel protein with a minimum of adjustable parameters. In addition, since each organ is described individually, one can predict their individual tissue protein PK, which is useful if trying to direct therapy to specific organs.

Although there has been great interest in the PK modeling of therapeutic proteins, there is another class whose PK has been almost completely ignored: the diagnostic enzymes such as alanine transaminase, aspartate aminotransferase, alkaline phosphatase, amylase, etc. Pathological disruption of normal cellular structure may result in the release of these cellular proteins to the interstitial space, with subsequent transport to the plasma. Because low concentrations of cellular enzymes are easily quantified via activity measurements, plasma enzyme values are widely used as routine screening tests for a variety of conditions as well as for evaluating the severity of specific disease states. These diagnostic enzymes are typically characterized by estimates of their “half-life”, based primarily on the time course of the fall in the plasma concentration following the resolution of the problem causing the enzyme elevation. Lindena et al. [9] have presented a remarkably thorough compilation of this older data, comparing the reported half-lives for man, dog and rat. These estimates are only approximate because of the lack of quantitative information about the ongoing release of the enzymes and/or recycling from the tissue space. Physicians tend to interpret plasma protein concentrations primarily as an indicator of a disordered rate of delivery to the plasma. Details of the PK: rate of release into the blood, time course and details of the extravascular distribution, and site and rate of removal usually are ignored. A quantitative description of the PK of these commonly measured plasma proteins, with an emphasis on the roles of distribution and elimination rates as determinants of plasma concentrations, should aid in the clinical interpretation of the time course of the plasma concentration.

In this review, we will first discuss the compartmental and PBPK approach to protein PK. We will show that, although the full PBPK approach is probably not feasible for most proteins, the use of a simpler physiological model, in which some organs are lumped together, is useful. We describe a new model that provides an accurate description of the plasma PK requiring, for a given species, only 2 adjustable parameters to characterize each protein. Plasma protein PK are quite simple and can be described by a variety of kinetic models. An important constraint on the model validity is its ability to describe the simultaneous tissue or lymph concentration, an additional constraint that is rarely discussed. We show that, in addition to describing the plasma concentration, this model is surprisingly successful at predicting the lymph and tissue concentration, a result that provides additional support for the validity of the model. Our model is much simpler and, therefore, more limited than some of the detailed PBPK models that have been developed for the monoclonals [8]. We believe that this simplicity and its ability to describe a wide range of proteins compensate for these limitations.

We will first discuss and illustrate compartmental and physiological PK modeling by applying it to albumin, the most extensively studied plasma protein (Section II). Then, in the following sections, this physiological model will be applied to the monoclonal antibodies (Section III) and the diagnostic enzymes (Sections IV – VIII). The resultant PK parameters for the 13 proteins investigated are summarized in Table 1 in terms of the compartmental parameters and in Table 2 in terms of the physiological parameters. The range of proteins analyzed is limited by the available PK data. The ideal would be human data for the plasma time course of an intravenous (IV) bolus of the purified human protein. For the proteins where these data do not exist, we assumed that data from studies in large mammals (e.g., dog, lamb, baboon) can be extrapolated to the human. Rat data is only sparingly employed because the extrapolation of rat PK data to humans is uncertain.

## Albumin pharmacokinetics: compartmental and physiological modeling

The distribution and catabolism of albumin has been extensively studied and will serve as our basis for developing the compartmental and physiological models applied to all the other proteins. Albumin capillary permeability must be very low in order to preserve the high plasma concentrations that maintain the colloidal osmotic pressure gradient between plasma and extravascular fluid. The permeability is not zero, however, and lymph drainage of the tissue space is required to maintain this concentration gradient. Controversy exists concerning whether this slow albumin leak is via caveolar vesicle transport versus through fixed pores or tight junctions [10]. Recent results showing that mice lacking endothelial caveolae have normal capillary albumin permeability [11] provide direct support for the pore mechanism. In addition, the proteins leak at rates are roughly inversely proportional to their molecular radius [12], a characteristic of molecules passing through pores rather than via transcytotic vesicle transport. An important question is whether this albumin leak represents a bidirectional diffusional permeability versus a unidirectional convective transport from the plasma to the tissue and return via lymph [13,14]. Although some quantitative measurements of simultaneous plasma and lymph protein PK are supportive of the convection mechanism [12,13,15], these results do not rule out a small diffusional component. It is assumed in the most recent protein modeling that the leak is entirely convective with no significant diffusional component [5,6,16], and we will make the same assumption.

**Figure 1.**
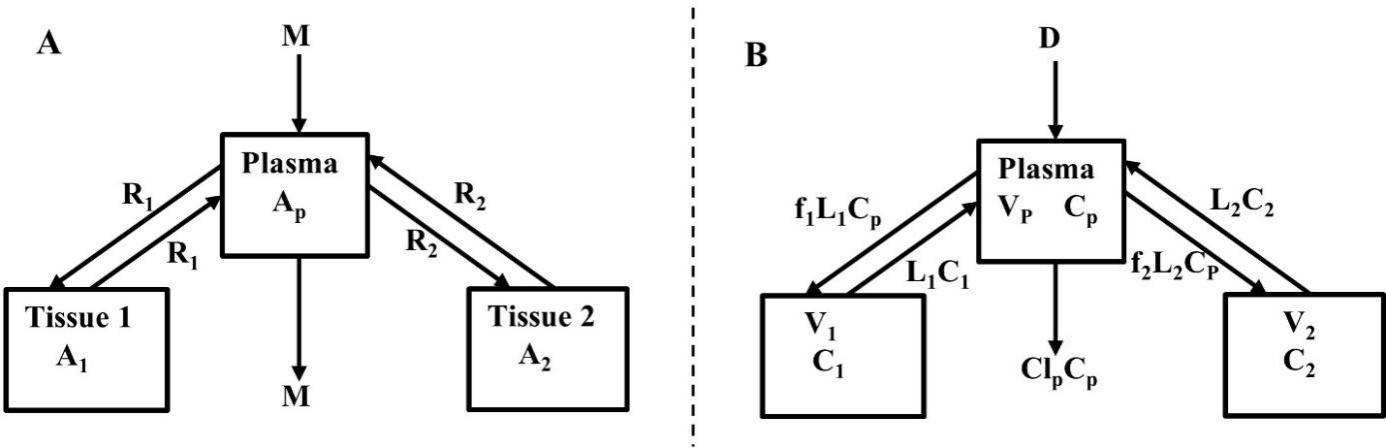
**A)** Beeken et al. steady-state kinetic model. **B)** Interpretation of the model in terms of one-directional convective filtration from plasma to the tissue and return via lymph flows to the plasma.

Beeken et al. [3] and Takeda and Reeve [4] have published similar compartmental PK human albumin analyses using the standard 3-compartment modeling approach. In the model of Beeken et al. diagramed in Figure 1A, the plasma albumin exchanges with two tissue compartments and the kinetics are characterized by 6 steady state constants: *M*, *A*_p_, *A*_1_, *A*_2_, *R*_1_, and *R*_2_. In the steady state, *M* equals the rate of synthesis which equals rate of catabolism, *A*_p_, *A*_1_, and *A*_2_ are the amounts in each compartment and *R*_1_ and *R*_2_ are the exchange rates. The pharmacokinetics of proteins has the simplifying characteristic that their distribution is limited primarily to the extracellular space. Thus, A_p_ is the amount in the plasma volume *V*_p_ and the plasma albumin concentration is *C*_p_ = *A*_p_/*V*_p_. Similarly, A_1_ and A_2_ are the amounts in the compartmental interstitial volumes [17]. These 6 parameters can be reduced to 5 by scaling them to the amount in plasma (*A*_p_) and expressing them in terms of the following five time constants (in units of minutes):


(1)

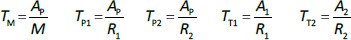



In the steady state case, when albumin is being synthesized at the same rate that it is removed by catabolism/excretion, *T*_M_ would be the time required to catabolize/excrete the initial amount in the plasma (=*A*_p_). In the non-steady state in which there is no new synthesis of albumin, *T*_M_ is the time for catabolism/excretion to lower the blood albumin concentration to 0.368 (=1/e) of its original concentration, assuming no tissue exchange. Similarly, *T*_P1_ and *T*_P2_ are the time constants for the fall in plasma concentration produced by filtration to the tissue, and *T*_T1_ and *T*_T2_ are time constants for the washout of the tissue compartments. All PK results are scaled to the human with an assumed plasma volume of 2800 ml.

**Figure 2.**
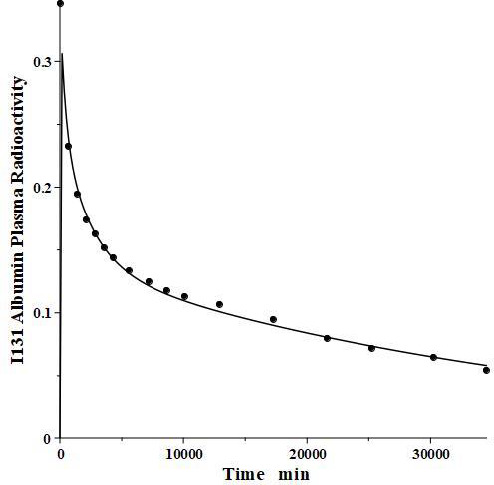
Comparison of PK compartmental protein model (line) for bolus IV human I^131^ albumin versus experimental data (solid circles) of Takeda and Reeve.

We have incorporated this compartmental model into a general computer routine using Maple (Maplesoft) with the 6 parameters determined by simple trial and error adjustments. As a test, Figure 2 shows the good agreement of the theoretical results (solid line) versus the independent experimental data of Takeda and Reeve [4] for the time course of plasma I^131^-albumin following a bolus input to the plasma, using the rate constants listed in Table 1, differing by only a few percent from those of Beeken et al. [3]. The metabolic time constant (*T*_M_) is 16,750 minutes (11.6 days). This is the hypothetical time constant for the exponential fall in the plasma concentration where the plasma albumin does not exchange with the tissue. The actual plasma PK, including tissue exchange, is multi-exponential and, therefore, cannot by described by a single time constant. However, because T_M_ is much longer than the other time constants, at long times when the plasma and tissue are approximately equilibrated the plasma PK has a time constant equal to (*A*_P_ + *A*_1_ +*A*_2_)/*M* =*T*_M_(1+*T*_T1_/*T*_P1_+*T*_T2_/*T*_P2_) (Figure 1A) or 26 days. The “half-time” required for the plasma concentration to fall to half its initial concentration is only about 2 days, reflecting primarily the time constants for distribution to the tissue (*T*_P1_, *T*_P2_).

For PBPK modeling, it is necessary to specify the physiological parameters that determine the protein PK (flow, convective transport, lymph flow, tissue volumes, etc.) for each organ. The obvious difficulty with this approach is the huge number of model parameters, most of which cannot be directly determined. For example, in the rat model of Shah and Betts [6], there are 16 organs, each described by 5 or more parameters, for a total of 90 parameters. PBPK modeling has been primarily useful for some small molecules for which the important parameters are simply the organ blood flow and volume, along with some other parameters that can be predetermined [18,19]. In marked contrast, protein PK depends on organ parameters such as capillary convective transport, lymph flow and the convective sieving factor for the protein, factors that, at present, cannot be accurately predetermined. In an animal model such as the rat, one can use the individual organ tissue concentrations to put some constraints on these parameters [6], but most remain just educated guesses. For the human, tissue measurements are not possible, severely limiting the value of a complete PBPK model.

Instead, we have developed the “lumped physiological” model shown schematically in Figure 1B. It is assumed that the “Tissue 1” compartment (Figure 1A) with the slow turnover time (*T*_T1_, Table 1) corresponds to the lumped tissues with relatively low lymph flow (e.g., skeletal muscle, skin, etc.) and the high turnover compartment (Tissue 2, *T*_T2_) represents the high lymph flow tissues (e.g., gastrointestinal tract). There are unidirectional convectional volume flows from plasma to each tissue space of *L*_1_ and *L*_2_ and equal lymph flows *L*_1_ and *L*_2_ returning to the plasma. In the convective flow from the plasma to tissue, the protein is sieved by factors of *f*_1_ and *f*_2_, so that the rate of transport from blood to tissue is equal to *f*_i_ *L*_i_ *C*_p_. There is no sieving of protein in the returning lymph with the lymph concentration equal to that in the tissue space so the rate of return to the blood is *L*_i_*C*_i_. *D* is the dose into the blood compartment and Cl_p_ is the plasma metabolic clearance (in units of ml/min).

The plasma protein pharmacokinetics is a function of just the 5 compartmental time constants (Eq. 1) and does not depend on how they are interpreted physiologically. However, to also model the lymph flow or the lymph protein concentration using the lumped model (Figure 1B). it is necessary to assume two additional variables that assign physical volumes to the tissue compartments (*V*_1_, *V*_2_). Using these volumes, the metabolic plasma clearance (Cl_p_), lymph flows (*L*_1_, *L*_2_) and sieving factors (*f*_1_, *f*_2_) in Figure 1B can be expressed as functions of the five compartmental time constants:


(2)

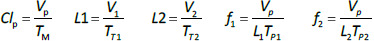



Alternatively, if the two lymph flows are known, then Eq. 2 can be used to calculate the volumes *V*_1_ and *V*_2_, given *T*_T1_ and *T*_T2_. The time dependence of the concentration in the plasma (*C*_p_(*t*)) and the two tissues (*C*_1_(*t*), *C*_2_(*t*)) is described by the 3 coupled differential equations:


(3)

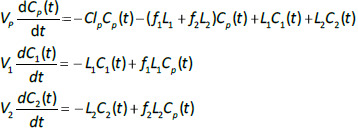



The advantage of the lumped physiological model (Figure 1B) is that it places major physiological limitations on the model parameters, i.e., there is not a near-infinite number of manipulations that can be employed to make the model fit the data. In our fitting of the data, we have made the following assumptions: 1) For a given species (e.g., human, dog, etc.), the value of the lymph flows *L*_1_ and *L*_2_ and tissue volumes *V*_1_ and V_2_ must be the same for all proteins studied in that species. 2) The capillary filtration “pore” must be roughly the same for the two tissue regions, implying the sieving factor *f*_1_ ≈ *f*_2_. 3) The values of f must be physiologically reasonable, i.e., *f* must be <=1 and, for a given species, proteins with similar molecular weights should have similar f, and f must decrease as the protein molecular weight increases. These assumptions dramatically simplify the model. Assuming that *L*_1_, *L*_2_, *V*_1_ and *V*_2_ have been previously determined for this species, in place of the 5 adjustable parameters in the general model, there are only two parameters that can be adjusted to fit different proteins for the same species: the plasma metabolic clearance (Cl_p_) and the sieving factor (≈*f*_1_ ≈ *f*_2_). In addition, for a given species, this sieving factor must decrease as the protein molecular weight increases. As shown in Table 2, we have been able to successfully fit the PK data for the 13 investigated proteins using these constraints on the model parameters.

The most questionable of these assumptions is the assumption that *f*_1_ ≈ *f*_2_. Zhao et al. [16] have used a similar two-compartment convective model to describe the plasma PK of a variety of monoclonal antibodies in 8 different animal species. They assumed that compartment 1 is “leaky” with a sieving coefficient f near 1, and compartment 2 is “tight” with an f near 0. The primary reason for this choice is to account for the liver, whose sinusoids are highly permeable (“leaky”) for most proteins. We disagree with this approach. The liver sinusoids are so highly permeable that the exchange of albumin between the plasma and liver interstitial space is flow-limited [20], that is, the sinusoidal exchange is so fast it is as if the sinusoidal barrier is not present. Thus, given the high liver blood flow, the liver interstitial space should equilibrate with the plasma within a few minutes. In the typical PK measurement, one waits for about 5 to 10 minutes after the bolus IV protein injection for plasma equilibration before taking the first plasma sample to follow the rate of decrease in concentration. Because of the flow-limited liver protein exchange, the liver will have completely equilibrated before the first sample is taken, and it will not contribute to the subsequent fall in plasma concentration. Thus, in our model, the liver is not included in the two tissue compartments in Figure 1B and the liver interstitial space is regarded as a component of the plasma volume *V*_p_. In all the other non-liver tissues, albumin is highly impermeable, as indicated by the fact that it exerts its theoretical colloid osmotic pressure across the capillary membrane, which is essential for fluid balance in all these tissues. Although the mechanism of the slow convective leak is poorly understood, whatever the mechanism, we think it is likely that, for the same species, the value of *f* should be roughly the same in all these non-liver tissues. It should be noted that plasma protein PK is quite simple, and any model with a few adjustable parameters will be able to fit the plasma concentration data. The ability of the model to also fit the experimental measurements of the lymph concentration places important additional constraints on the model and is a factor that we have emphasized in this review. The liver does complicate the interpretation of the lymph measurements. Since the liver lymph flow is relatively large, about 20 % of the thoracic duct flow [21], liver lymphatics, which are neglected in our model, should make a significant contribution. However, as shown below, lymph concentration measurements are highly variable and deviations of 20 % are not considered significant.

The lumped tissue volumes *V*_1_ and *V*_2_ and the corresponding lymph flows *L*_1_ and *L*_2_ (Eq. 2) cannot be directly measured experimentally. They are simply model parameters that are empirically adjusted to fit, at least, the following three experimental observations. The total lymph flow relation:


(4)

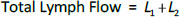



The steady-state tissue concentrations are described by (*C*_1_/*C*_p_)_ss_ = *f*_1_ and (*C*_2_/*C*_p_)_ss_ = *f*_2_, so that the experimentally measurable steady-state total lymph protein concentration relative to the plasma concentration (*C*_Lss_/*C*_P_) is:


(5)

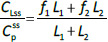



Finally, the steady-state extravascular volume of distribution (*V*_ECF_), which can be determined experimentally from the PK following a bolus injection [18], is described by:


(6)

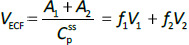



where *A*_1_ and *A*_2_ are the steady-state amounts in the tissue compartments (Figure 1A). For each experimental animal model (e.g., human, dog, etc.), *V*_1_ and *V*_2_ were adjusted to satisfy these three experimental observations (Eqs. 4-6).

For the human, we estimated that *V*_1_/*V*_p_ = 1.3 and *V*_2_/*V*_P_ = 0.4, which produces albumin sieving factors (Eq.2) *f*_1_ =0.73 and *f*_2_=0.725. These values satisfy the 3 conditions (Eqs. 4-6) for albumin. The corresponding lymph flows (Eq. 2) are *L*_1_ = 0.87 ml/min and *L*_2_=2 ml/min for a total lymph flow (Eq. 4) of 2.8 ml/min. Although there is no measurement of normal human total lymph flow (thoracic duct plus right lymph duct), this result is similar to the few available normal human thoracic duct measurements [22,23]. The corresponding theoretical steady state albumin lymph/plasma ratio (Eq. 5) is 0.725, identical to that reported by Witte et al. [24] for human thoracic duct lymph. Finally, *V*_ECF_ (Eq. 6) is 3479 ml, similar to the value of 3250 determined from the PK analysis following the bolus IV injection (Figure 2). These physiological parameters for albumin and all the other proteins studied are summarized in Table 2. Tissue region 1, which has a larger volume (*V*_1_/*V*_p_ = 1.3) and slower turnover time (*T*_1_= 4182 minutes), presumably corresponds to the lumped tissues with relatively low lymph flows such as the skin, subcutaneous and skeletal muscle tissues, while tissue region 2, with the smaller volume (*V*_2_/*V*_P_ = 0.4) and faster turnover time (*T*_2_=557 minutes), presumably corresponds to the GI tract which is known to have a relatively high lymph flow rate [13]. The physiological model fit to the albumin data is identical to the compartment fit (Figure 2) because we have defined the albumin physiological parameters (Table 2) directly in terms of the previously determined compartmental parameters (Table 1, Eq. 2).

The focus of this review is on the PK, not on the detailed metabolic mechanisms. The main assumption of both the compartmental and physiological model is that the catabolism or clearance occurs from the plasma space, i.e., it is proportional to the plasma concentration. Both albumin and immunoglobulins are known to be metabolized by endosomal metabolism that is moderated by FcRn binding [5,7,25]. Since this endosomal space is usually assumed to be located in the vascular endothelium [5,7] which is in contact with the blood, this is consistent with the metabolic assumption. Similarly, the diagnostic enzymes are either metabolized by the liver or cleared by the kidney (Table 1), both of which are also proportional to the plasma concentration.

## Monoclonal Antibodies

**Figure 3.**
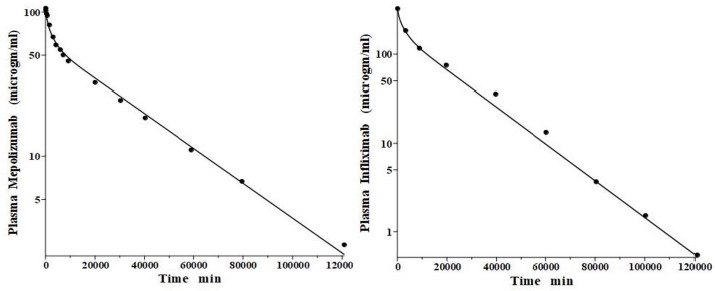
Comparison of PK physiological protein model prediction (line) following IV bolus monoclonal antibody injection in the human versus experimental data (solid circles). Left panel: mepolizumab; Right panel: Infliximab. Only one parameter (plasma clearance) differs for the two model fits.

IgG and albumin are the classical markers of capillary protein permeability. With a molecular weight about twice that of albumin, IgG has a lower filtration coefficient and a corresponding lower steady-state lymph concentration, about 60 % that of albumin [24,26,27]. The recent development of monoclonal antibodies for therapeutic purposes has resulted in an extensive investigation of the human PK of these proteins and has been the subject of several recent reviews [28-30]. Figure 3 (left panel) shows the physiological model fit to the data of Smith et al. [31] for the plasma disappearance curve of an IV bolus infusion of the monoclonal antibody mepolizumab. As discussed, since the lymph flows (*L*_1_, *L*_2_) and tissue volumes should be equal to those determined above for albumin in the human, there are only two parameters that can be adjusted to fit this data, the plasma clearance (Cl_p_) and the filtration constant *f* (≈ *f*_1_ ≈ *f*_2_), which should be less than then the albumin value of *f*=0.73 since mepolizumab has a molecular weight 2.2 times that of albumin. The physiological model fit in Figure 3 is in accord with these restrictions, having an *f*_1_ = 0.367 and *f*_2_ = 0.361, half that for albumin, and the same lymph flows and tissue volumes used for albumin (summarized in Table 2). As predicted by the physiological model, the PK of another monoclonal, infliximab [32], was accurately described by adjusting only Cl_p_, with all the other parameters identical to those for mepolizumab since it has a nearly identical structure and, therefore, should have identical filtration constants (data in Table 2, Figure 3, right panel).

**Figure 4.**
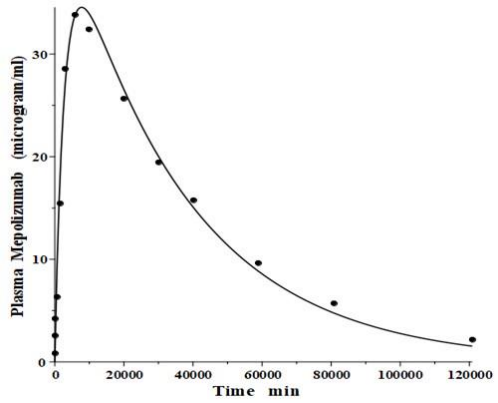
Comparison of physiological protein model prediction (line) of the plasma concentration following a subcutaneous bolus Mepolizumab injection in the human versus experimental data (solid circles) using the identical model parameters used for IV input in Figure 3.

In addition to the IV pharmacokinetics for mepolizumab, Smith et al. [31] also described the plasma concentration following a bolus subcutaneous injection. It is of interest to determine if this data is consistent with the predictions of the physiological model (Figure 1B) using parameters identical to those determined from the IV input data (Figure 3, Table 2). As discussed above, the slower lymph flow, longer turnover time region *V*_1_ presumably corresponds roughly to that of the subcutaneous tissue. Thus, the subcutaneous dose of mepolizumab was presumably injected into a tissue with kinetics similar to that of *V*_1_. Figure 4 compares the experimental plasma concentration following the subcutaneous injection versus the physiological model prediction for a bolus dose into tissue V_1_ using the identical model parameters obtained from the IV input (Figure 3), with no adjustable parameters. The agreement between model prediction and experimental data is remarkably good, given the simple two-tissue compartment model assumption. This result provides additional support for the physiological model (Figure 1B) with its corresponding quantitative implications for the lymph flow rates (Eq. 2).

## Amylase and lipase

Lipase and amylase, two enzymes used to assess the pancreatic injury, are unique among the proteins listed in Table 1 in that they are cleared primarily by the kidney. Junge and colleagues [33,34] have elucidated their pharmacokinetics in the rat. Both are rapidly cleared from the blood with a single compartment, one-exponential metabolic time constants *T*_M_ of 31 min and 26 min for amylase and lipase, respectively. About 70 % of this clearance is renal, with both enzymes having surprisingly high glomerular filtration rates, roughly 12 % of inulin. About 84 % of the filtered amylase appears in the urine as an intact, enzymatically active protein, while nearly all the filtered lipase is metabolized by the kidney with negligible amounts of active enzyme appearing in the urine.

**Figure 5.**
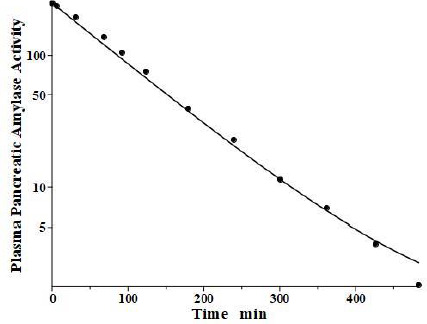
Comparison of PK physiological protein model prediction (line) following IV bolus of baboon purified pancreatic amylase versus the experimental data in baboon (solid circles). The activity is plotted with the basal background subtracted.

Levitt and colleagues [35,36] have reported a detailed analysis of the pharmacokinetics of amylase in the baboon. They determined the plasma kinetics of enzyme activity following a bolus IV injection of either salivary or pancreatic amylase, both of which were rapidly cleared with nearly identical kinetics and could be described by a single exponential with a *T*_M_ of about 100 min. Complicated PK modeling is superfluous for proteins with T_M_ much shorter than the plasma-tissue exchange time constants (*T*_P1_, *T*_P2_, *T*_T1_, *T*_T2_) because these proteins, effectively, are limited to the plasma space and should have one compartment, single exponential, kinetics with *T*_M_ equal to 1/slope of the straight line in the log plot. Since *T*_M_ is uniquely determined by the plasma volume and the clearance (Eq. 2), the clearance is the only adjustable parameter. However, it is of interest to check whether a single exponential is consistent with our protein model. Figure 5 shows the model fit assuming amylase f_1_ and f_2_ equal to those of albumin, which has a similar molecular weight and *L*_1_ and *L*_2_ for the baboon assumed identical to the human (Table 2). Only the last experimental data point deviated from this model result. Since the experimental data is for the “excess” amylase above the initial basal value, just a small drift (≈ 2 units/ml) in basal amylase could account for this small deviation. Or, if the data are correct, it would suggest that the tissue-plasma exchange of amylase is significantly slower than albumin. Note, the values of the parameters *f*_1_, *f*_2_, *L*_1_, and *L*_2_ listed in Table 2 for amylase and lipase only indicate that they are compatible with the plasma PK, but they were not directly determined because of the short metabolic time constant.

Although there are no equivalent large animal kinetic studies of lipase, we have assumed identical kinetics for lipase in Table 1 because of the nearly identical lipase and amylase kinetics in the rat [33].

## Alanine transaminase (ALT)

Alanine transaminase, also known as alanine aminotransferase or glutamic pyruvate transaminase, is released from a variety of tissues under pathological conditions. Its primary site of elimination is probably the liver. At least three different liver systems are involved in protein catabolism: the sinusoidal cells, the Kupffer cells and the asialoglycoprotein receptor (see below). Kupffer cells are macrophages that line the surface of the sinusoids [37] and their metabolic activity for different enzymes can be quantitated by competition experiments [38]. Because the Kupffer cells usually have higher rates of metabolism than the sinusoidal cells, the proteins they catabolize usually have shorter time constants. ALT is not metabolized by Kupffer cells, consistent with its relatively long lifetime [38].

**Figure 6.**
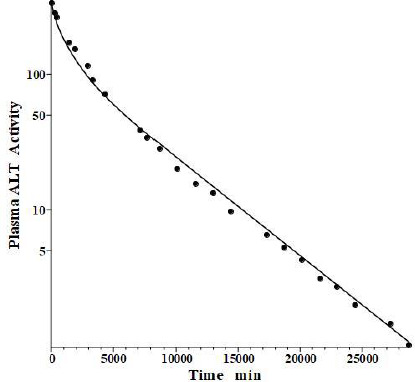
Comparison of PK physiological protein model (line) for ALT bolus IV injection versus experimental data (solid circles) in dog.

Fleisher and Wakim [39] determined the dog disappearance kinetics of IV injections of ALT purified from either dog liver or heart (no significant differences). Importantly, they also reported simultaneous measurements of thoracic duct lymph ALT concentrations, which, as discussed above, can be used to estimate the tissue volumes. Using these data, we estimated the dog values of tissue volumes of *V*_1_/*V*_p_ = 1.0 and *V*_2_/*V*_p_ = 0.3, differing slightly from the values determined above for the human. These values then allow us to relate the compartmental model time constants to the filtration coefficients and lymph flows using Eq. 2. Figure 6 shows the good fit of the protein model to the plasma concentration following the IV bolus injection, using the PK data listed in Tables 1 and 2. The metabolic time constant (*T*_M_) is 2949 min (2 days), 5.5 fold faster than albumin. The corresponding *f*_1_=0.53, *f*_2_=0.535. The estimated lymph flows are *L*_1_ =1.2 and *L*_2_ =2.7, for a total lymph flow = 3.9 ml/min, similar to the 3.5 ml/min value reported in resting conscious dogs [40]. (Note, all values reported in this review have been extrapolated to the 70 kg human).

The simultaneously measured thoracic duct lymph ALT concentration had relatively large variations between dogs [39]. The model total lymph concentration is given in terms of the total lymph drainage of the two tissue compartments (Figure 1B):


(7)

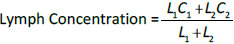



**Figure 7.**
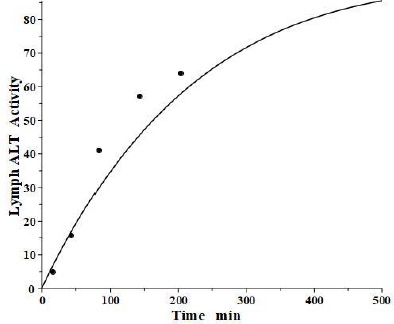
Comparison of PK physiological protein model lymph thoracic duct ALT concentration following IV bolus injection in dog (line) versus experimental data (solid circles) of Fleisher and Wakim.

Figure 7 shows the relatively good model fit obtained for the lymph data for one dog. These tissue volumes and lymph flows should be regarded as only rough approximations because of the large variation between dogs and the assumption that there are only two tissue compartments. However, the finding that the thoracic duct data is in general agreement with the model predictions with reasonable physiological parameters is encouraging and provides additional support for the physiological model (Figure 1B), with the parameters summarized in Table 2.

## Aspartate aminotransferase (AST) and creatine kinase (CK)

**Figure 8.**
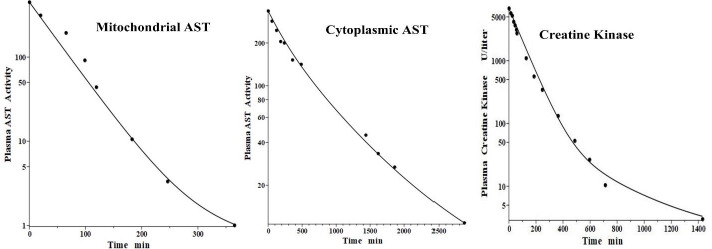
Comparison of PK physiological protein model prediction (line) following IV bolus dose of mitochondrial (left panel) or cytoplasmic (middle panel) AST and creatine kinase (right panel) versus experimental data (solid circles) in dog.

Aspartate aminotransferase, also known as glutamic-oxalacetic transaminase, is a clinical marker for hepato-cellular injury secondary to a variety of inflammatory conditions. There are two forms of the enzyme found in plasma, the “cytoplasmic” and “mitochondrial”, with similar structures but markedly different pharmacokinetics [41,42]. Fleisher and Wakim [41] investigated the dog PK for both forms of purified dog liver ALP. Since AST has a molecular weight similar to ALT, it should have the same *f*_1_ and *f*_2_ and lymph flows *L*_1_ and *L*_2_ as those determined above in the dog for ALT. Thus, there is only one adjustable parameter: Cl_p_. Figure 8 and Table 2 show that, indeed, both cytoplasmic and mitochondrial plasma AST concentration following an IV bolus could be successfully fit by the protein model using the previously determined dog ALT *f*_1_, *f*_2_, *L*_1_ and *L*_2_. The ability to fit this novel data using only one adjustable parameter dramatically illustrates the advantage of using this physiological model. Mitochondrial AST is metabolized by the liver Kupffer cells and, as is characteristic of this mechanism, has a very high metabolic rate, with a *T*_M_ of only 50 minutes. Cytoplasmic AST is metabolized primarily by the liver sinusoidal cells and has a *T*_M_ of 670 minutes, 13-fold slower than mitochondrial AST.

Creatine kinase (CK) is another Kupffer-metabolized protein [38]. Aktas et al. [43] have determined the plasma kinetics in the dog following IV injections of CK purified from dog muscle homogenates. CK should have the same dog *L*_1_ and *L*_2_ as ALT, but since it has about twice the molecular weight of ALT, it is expected to have smaller filtration constants and, therefore, there are two adjustable parameters: Cl_p_ and *f*. Consistent with this, the plasma PK data are well fit by the model (Figure 8, right panel) with *f*_1_ = *f*_2_ =0.37, 30 % smaller than ALT and AST, and the same dog *L*_1_ and *L*_2_ as ALT and AST (Table 2). As expected for liver Kupffer metabolism, it has a relatively short metabolic time constant (*T*_M_) of 88 min (Table 1).

## Lactate dehydrogenase (LD)

Lactate dehydrogenase is a tetramer constructed from different numbers of the two monomers called H (heart) and M (muscle), resulting in 5 different isoenzymes: LD1 (H_4_), LD2 (H_3_M), LD3 (H_2_M_2_), LD4 (HM_3_) and LD5 (M_4_) [44]. The predominant diagnostic isoenzymes are LD1 which increases in acute myocardial infarction and LD5, which is increased in skeletal muscle (e.g., muscular dystrophy) and liver pathologies [44]. LD5 is metabolized predominantly by liver Kupffer cells and, accordingly, should have a relatively short metabolic time constant. In contrast, LD1 is not metabolized by Kupffer cells and should have a corresponding slower metabolic rate [38].

**Figure 9.**
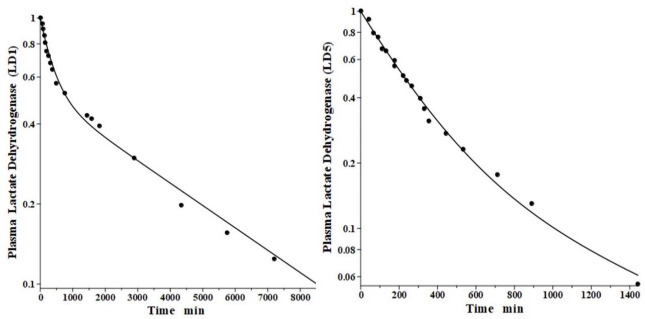
Comparison of PK physiological protein model prediction (line) following IV bolus injections in the lamb of the sheep purified protein lactate dehydrogenase isoenzymes LD1 (heart) (left) and LD5 (skeletal muscle) (right) versus experimental data (solid circles).

Boyd has reported the plasma kinetics in lambs following IV bolus injections of LD1 (purified from sheep heart) and LD5 (purified from sheep skeletal muscle). We assume the tissue space volumes are identical to those determined for the dog (*V*_1_/*V*_p_ = 1.0 and *V*_2_/*V*_p_ = 0.3). As shown in Figure 9, both forms were adequately fit using this convection model with the corresponding *f*_1_ = *f*_2_ =0.53, and lymph flows *L*_1_=3.85 and *L*_2_ = 1.35 (Table 2). These results are more uncertain than the dog or human because there are no lamb lymph protein data to constrain them. These lymph flows (total flow = 5.2 ml/min/70 kg), which are significantly larger than those of dog or human are consistent with the high values reported for lamb thoracic duct [45]. As expected for these two molecules with similar structures, they were fit with identical values of *f*_1_, *f*_2_, *L*_1_ and *L*_2_. The *T*_M_ for LD5 (Kupffer cell metabolism) is 465 minutes, more than 7 times shorter than the LD1 *T*_M_ of 3418 minutes (Table 1).

## Alkaline phosphatase (ALP)

**Figure 10.**
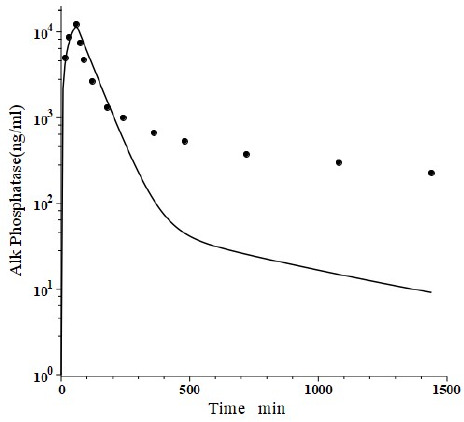
Comparison of PK physiological protein model prediction (line) following a 60 min IV infusion of recombinant alkaline phosphatase (recAP) in the human versus experimental data (solid circles). The human plasma-tissue exchange parameters (*f*_1_, *f*_2_, *L*_1_, *L*_2_, Figure 1B) were assumed to be identical to those for monoclonals and only the metabolic clearance (Cl_p_) was adjusted to try to fit the early time data.

Peters et al. [46] have developed a human recombinant alkaline phosphatase (recAP) as a potential therapeutic agent. This human chimera is constructed from 414 amino acids from the intestinal plus 70 amino acids from the “crown” domain of placental alkaline phosphatase [47]. Since the recAP PK is nearly identical to purified bovine intestinal alkaline phosphatase [48], it can be assumed that the recAP PK is representative of human intestinal alkaline phosphatase (IAP). Studies of recAP provide some of the best available human protein enzyme PK studies because the protein is readily distinguished from the background endogenous enzyme. Since recAP has a molecular weight nearly identical to that of IgG, it should have the same *f*_1_, *f*_2_, *L*_1_ and *L*_2_ that were previously determined for IgG in humans, leaving only one adjustable parameter, Cl_p_. Figure 10 shows an attempt to fit the recAP kinetics (solid circles) following a 60 min constant IV infusion of 1000 U/kg in humans with the convective model (Figure 1B) using the IgG *f*_1_ and *f*_2_, and the human lymph flows (*L*_1_ and *L*_2_), adjusting only Cl_p_ to fit the early time data. It can be seen that recAP has marked biphasic kinetics that is very poorly fit by the model. No reasonable variation of the model parameters can fit the anomalously rapid, initial, distribution phase of the enzyme. Clearly, a major modification of the protein model in Figure 1B is required for recAP.

Studies in the rat, both in vivo well as in isolated liver perfusion studies, have provided a mechanistic explanation of this striking biphasic kinetic behavior [49-52]. Alkaline phosphatase is unique among the proteins under study (Table 1) in that it is a glycoprotein whose elimination involves binding to the mannose and galactose (also known as asialoglycoprotein) liver receptor. The rates of both the early (distribution) and late (elimination) phases of the alkaline phosphatase curve are dramatically reduced by the addition of asialofetuin, an inhibitor of the asialoglycoprotein receptor. The rat results suggest that the early, extremely rapid phase of the kinetics results from alkaline phosphatase attachment to the asialoglycoprotein receptor, most of which is then taken up by the liver cell and recycled back to the plasma where it is released intact, with little metabolism.

**Figure 11.**
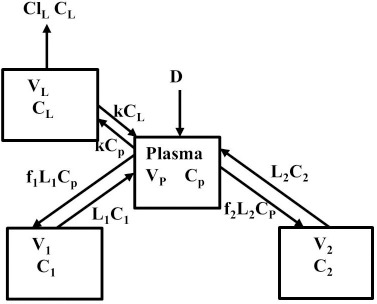
Modified physiological protein model with the addition of a recycling liver compartment with metabolic clearance Cl_L_ from the liver compartment.

**Figure 12.**
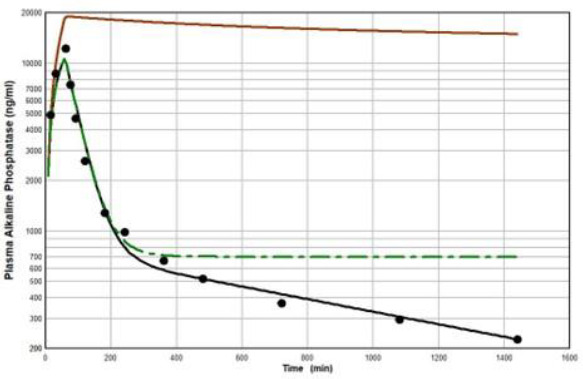
Comparison of physiological liver recycling model (Figure 11) prediction (black line) following a 60 min IV infusion of recombinant alkaline phosphatase (recAP) in the human versus experimental data (solid circles). The red line is for the case where the liver exchange rate (*k*, Figure 11) is set to zero. The green dashed line is for the case where the liver metabolic clearance (Cl_L_) is set to zero.

Figure 11 diagrams a modification of the protein physiological model with the addition of another compartment (*V*_L_, *C*_L_) in an attempt to account for this additional recycling liver component. The rate constant *k* characterizes the recycling rate between the plasma and liver compartment, and *V*_L_ is the equivalent volume (i.e., binding capacity) of this compartment. Note that the metabolism now occurs from the liver compartment and is proportional to *C*_L_ and is characterized by metabolic liver clearance (Cl_L_). Although there are 7 PK parameters in Figure 11, only 3 of them are adjustable (*k*, *V*_L_, Cl_L_) because *f*_1_, *f*_2_, *L*_1_, and *L*_2_ should have the same values as the human monoclonal antibody PK. As shown in Figure 12, this modified model, with these limiting conditions, accurately simulates the human biphasic kinetics of recAP (using model parameters listed in Table 2). The liver compartment *V*_L_ has a very large volume (e.g., binding capacity), 26 times that of the plasma volume, and rapidly exchanges (*k*) with the plasma (50 times the rate of plasma to tissue transport). The fit is not perfect, which is not surprising given the complexity of liver recycling and metabolic processes. The red line shows that when the liver exchange rate *k* is set to zero, there is a dramatic reduction in both the clearance and tissue distribution, very similar to what is observed in the rat when the asialoglycoprotein receptor is competitively inhibited by asialofetuin [49,51]. The green dashed line (Figure 12) shows that setting the liver metabolic clearance (Cl_L_) to zero has only a minor effect on the early phase kinetics, which is dominated by the distribution into the liver compartment.

An interesting test of this PK model is to see if it can mimic the well-established, transient clinical elevations in plasma intestinal alkaline phosphatase (IAP) that follow the ingestion of a high-fat (but not protein or carbohydrate) meal [53]. There is a large individual variation in these fluctuations, with those who are red blood cell B or O antigen secretors having much greater IAP release. Domar et al. [53] have quantitated these fluctuations following a standard high-fat meal. Interestingly, they have shown that the baseline (fasting) IAP increases proportionally to the peak increase and that secretors with high fat-stimulated values have fasting IAP about 10 times greater than non-secretors with small fluctuations, strongly suggesting that the intestinal IAP release following a fatty meal is the sole supplier and determinant of plasma IAP.

**Figure 13.**
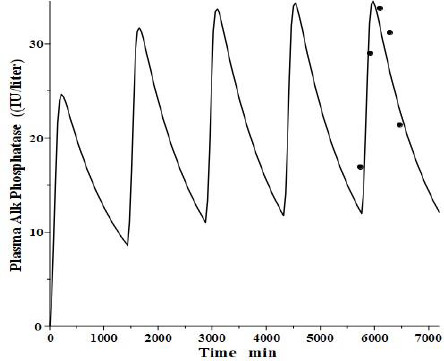
Comparison of physiological liver recycling model (Figure 11) simulation of the intestinal release of intestinal alkaline phosphatase following a high-fat meal every 24 hours (line) versus the experimental data of Domar et al. (solid circles).

We tried to fit the data of Domar et al. [53] with the modified PK model (Figure 11) using the model parameters determined above for reCAP, which is representative of IAP. We assumed that following a high-fat meal, IAP was released into the tissue volume *V*_2_ (Figure 1B), which, as discussed above, presumably corresponds to the GI tract, at a constant rate for 120 minutes, simulating the release of IAP into the intestinal interstitial space following a high-fat meal. In order to compare the results with that of Domar et al. for subjects that have established steady-state IAP levels, this 120-minute input was repeated at 24-hour intervals until a steady state was established. Figure 13 shows the comparison of our PK model predictions to the clinical data of Domar et al. [53] for one of their subjects (there was a large subject-to-subject variation). Given the crudeness of our model, the agreement with the data is relatively good. Of special significance is the fact that both the size of the IAP fluctuations and the corresponding basal fasting value (minimums in the plot) are in good agreement with the clinically observed values. This strongly suggests that the fasting value is entirely the result of the amount of IAP released during fatty meals. For the subject in Figure 13, a total of 360 IU was released with each meal, quantitating the daily human IAP release.

There are at least 4 gene loci coding 4 different tissue-specific alkaline phosphatase isoenzymes: 1) intestine, 2) liver/bone/kidney, 3) placenta, and 4) germ cell [49,54]. Clinical observations indicate that the intestinal form has the shortest half-live (most rapid clearance) placental the longest, with the liver/bone/kidney isoenzyme intermediate, a finding consistent with PK measurements in the rat [49]. Based on the PK model developed for recAP (Figure 11), one would predict that both the early phase (recycling) and late phase (metabolism) of the biphasic kinetics should be proportional to the affinity for the asialoglycoprotein receptor, which depends on the number of glycan chains and their degree of sialyation. This is consistent with the low clearance of the placental alkaline phosphatase, which has only one glycan chain, of which more than 50 % are sialyated [55].

**Figure 14.**
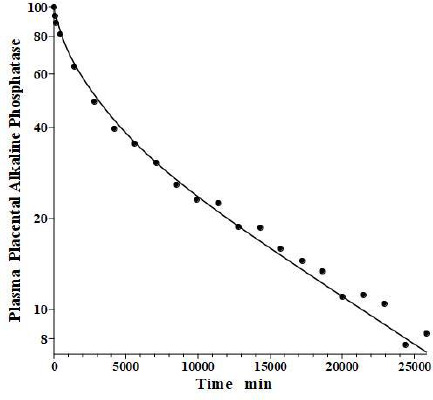
Comparison of standard physiological protein model (Figure 1B) prediction (line) following IV bolus injection of human purified placental alkaline phosphatase versus experimental data (solid circles).

In an older study, Clubb et al. [56] determined the PK of partially purified human placental alkaline phosphatase (PAP) following a bolus IV dose in humans, using the heat stability of PAP to distinguish it from the endogenous background plasma alkaline phosphatases. Because of the very low glycosylation of PAP, the liver recycling should be negligible, and we attempted to fit the PAP PK with the standard protein model (Figure 1B) using the monoclonal antibody *f*_1_ and *f*_2_, and the human lymph flows *L*_1_ and *L*_2_, with Cl_p_ as the only adjustable parameter. As can be seen in Figure 14, this fit is excellent, indicating that PAP is in the same class as all the other proteins in Table 2 and that IAP is the exception.

## Clinical relevance

The primary aim of this paper was to demonstrate that the distribution and elimination rate of an infused bolus of a wide variety of plasma proteins can be described by a relatively simple physiological PK model. Since all studies were carried out in healthy humans and animals, the data provided are normal values. From the medical care standpoint, the important question is to what extent can alterations in these normal distribution and elimination kinetics produce clinically relevant but frequently unrecognized changes in plasma protein concentrations. The answer to this question, which is complicated and different for each protein, is the topic of a subsequent paper. However, some generalities include the following.

Depending on the protein, either increases or decreases in elimination rate could cause alterations in the plasma value that could be of clinical importance. A clear-cut example of such an elimination defect is the formation of globulin-bound enzymes (“macro enzymes”), an anomaly reported for most commonly measured diagnostic plasma enzymes. The increased molecular size of these macroenzymes dramatically slows their catabolic rate in the liver or glomerular filtration rate (lipase, amylase) such that serum concentration rises roughly in proportion to the fraction of enzyme that exists in the bound form. A disease of the eliminating organ also may cause serum enzyme elevations. The most obvious example is kidney disease; up to three-fold elevations of the two enzymes cleared by the kidney, lipase and amylase, can be observed in renal insufficiency [57,58]. Similarly, hepatic injury, e.g., carbon tetrachloride-induced hepatic necrosis in rats, dramatically reduced the clearance of infused AST [42]. Thus, the elevated plasma level of the aminotransferases characteristic of hepatic injury reflects, in part, decreased elimination in addition to the increased release. Another example of the effect of decreased liver clearance is the striking increases in alkaline phosphatase produced by circulating inhibitors of the galactose (asialoglycoprotein) receptor, as postulated in a recent paper [59]. As discussed above, the degree of sialyation of glycoproteins has a dramatic effect on their elimination rates. Since the desialylation of circulating proteins is enhanced in various disease states, e.g., hyperthyroidism [60], this activity would be expected to have a major effect on the serum glycoprotein protein concentration.

Hypoalbuminemia is a common finding in a wide variety of disease states, and this finding is associated with a poor prognosis [25]. While clinicians commonly attribute these low serum albumin values to decreased synthesis (“malnutrition”, etc.), multiple studies have shown that, with the exception of cirrhosis and kwashiorkor, it is actually increased elimination that is the major cause of hypoalbuminemia [25]. This enhanced elimination may be due to increased activity of the normally catabolizing organs or an unusual rate of loss of albumin into the gut lumen or urine [61]. The development of IgG monoclonal antibody therapies has stimulated interest in the elimination rate of these compounds [28-30]. The frequency of IgG dosing required to maintain a therapeutic plasma concentration as a function of elimination rate and trough concentration measurements have become routine clinical assays for some monoclonal antibodies. Alteration of the molecular structure of selected monoclonal antibodies has been used to reduce the elimination rate and hence prolong effectiveness [62].

Much of the emphasis in this PK modeling discussion was focused on the kinetics of exchange and distribution with the extravascular space. However, at homeostasis, the rate of delivery to and return from the extravascular pools are equal, hence the plasma concentration is independent of the rate of distribution and depends only on the rates of synthesis and elimination. The rates of exchange with the extravascular pools as well as the size of the pool, do influence non-steady state plasma concentrations, such as the timing and magnitude of peak plasma concentrations following periodically administered monoclonal antibodies or the rate of rise and fall of liver enzymes after a brief insult. The appreciable extravascular distribution of proteins (e.g., 1.24 times the plasma volume for albumin at homeostasis) buffers the rate of change in plasma protein in the non-steady state, hence, influencing clinically important values such as the apparent half-time of various proteins.

## Summary

The three-compartment model shown in Figure 1A is the standard 3-exponential, 6-parameter description routinely used to describe plasma PK. We have interpreted this model in terms of a lumped physiological model (Figure 1B) in which the exchange between the blood and tissue is purely convective, dependent on the lymph flows *L*_1_ and *L*_2_ and filtration coefficients *f*_1_ and *f*_2_. For a given species, we first estimate the four adjustable model parameters L_1_, L_2_ and tissue volumes *V*_1_ and *V*_2_. Assuming that these parameters are the same for any protein in that species implies that each protein is uniquely characterized by only 3 parameters: the clearance Cl_p_, and *f*_1_ and *f*_2_. Imposing the additional constraint (satisfied by the results in Table 2) that *f*_1_ ≈ *f*_2_, effectively reduces it to two adjustable parameters.

Interpreting these parameters in terms of the physiological convection model (Figure 1B) has two important advantages. First, as discussed above, it puts strong bounds on the values of the parameters. For example, the human PK of an arbitrary protein should have the same values of *V*_1_, *V*_2_, *L*_1_ and *L*_2_ determined in Table 2 and since *f*_1_ and *f*_2_ can be estimated from the molecular weight, there is actually only one primary adjustable parameter, the plasma clearance (Cl_p_), in place of the 5 parameters of the compartmental model (Figure 1A, eq. 1). Secondly, it allows one to use the plasma PK to predict the tissue and lymph protein kinetics: 1) the total lymph flow (eq. 4), 2) the steady-state lymph/plasma protein ratio (eq. 5), 3) the simultaneous plasma and lymph protein concentration following a bolus ALT IV injection (Figure 7), 4) the plasma concentration following a subcutaneous monoclonal antibody injection (fig 4), and 5) the plasma concentration of IAP following a high-fat meal (Figure 13). The validity of these predictions provides the strongest available evidence in support of this simple physiological model.

As discussed, one of the assumptions of the model is that, for a given species, the sieving coefficient f should decrease as its molecular size increases. The results in the human are consistent with this assumption with an *f* = 0.73 for albumin (mol wt= 66,500) and a smaller *f* = 0.367 for the larger monoclonal antibodies (mol wt = 149,000) and similar size alkaline phosphatase (mol wt = 140,000). The dog results are also consistent: *f*= 0.53 for alanine transaminase (MW = 54,600) and similar-sized mitochondrial and cytoplasmic aspartate aminotransferase (MW 47,000) and *f* = 0.37 for the larger creatine kinase (MW = 81,000). Although one could use these results to try to estimate an “equivalent capillary pore radius” [63], the data are too limited to put much confidence in these results. Note that these results also suggest that the equivalent pore radius in the human is larger than that in the dog.

This review is the first that we are aware of that uses a simple physiological model to describe a comprehensive framework for cataloguing the results of protein PK studies (Table 2). The parameters are consistent with the constraints placed on them by the model, i.e., the same *V*_1_, *V*_2_, *L*_1_ and *L*_2_ for all proteins in the same species, and f decreases as molecular weight increases. Even considering species variations, the plasma clearance (Cl_p_) is the main PK determinant, varying by more than 400-fold, with the other parameters varying by about 2-fold.

The one protein that did not fit this simple convection model was the intestinal isoform (recAP) of the glycoprotein ALP. The recAP PK is dramatically biphasic. In the first (distribution) phase, the plasma concentration falls to about 5 % of its initial value in 4 hours (see Figure 10). Since an impossibly large space of about 24 liters would be required to account for such a distribution, it appears that its initial disappearance phase is attributable almost entirely to receptor binding with rapid recycling in the liver. Metabolism is negligible during this distribution phase (Figure 12). The second slow metabolic phase only becomes apparent at long time periods, and the 24-hour data used in Figure 12 may not be long enough for an accurate estimate of the metabolism. This result illustrates one of the main advantages of using a physiological model. Although one can always fit the data with a compartment model with its 5 adjustable parameters, using the much more constrained physiological model (2 adjustable parameters) can provide unequivocal evidence that the model is wrong and that the compound under study must belong to a significantly different PK class.

In going to the simplified 2 tissue compartment lumped physiological model, one gives up the potential of predicting the detailed individual tissue PK in the 13 or more organs of the full PBPK model. However, given the limited knowledge, at least in the human, of each organ’s lymph flow, volume, and sieving coefficient, this is more of a theoretical than a practical limitation. The two lumped tissue compartments (*V*_1_ and *V*_2_) in our simplified model are surprisingly good at simulating tissue input. For example, the plasma concentration following a subcutaneous injection is well simulated by injection into the slow turnover *V*_1_ compartment (Figure 4) and the plasma concentration following gastrointestinal tissue input can be predicted by input into the fast turnover *V*_2_ compartment (Figure 13). We believe that the physiological model’s simplicity and ability to describe the plasma and lymph concentration for a wide range of proteins compensates for its theoretical limitations.

## Figures and Tables

**Table 1.** Summary of protein pharmacokinetics expressed in terms of the 5 compartmental time constants corresponding to the steady state parameters of Figure 1A. All results are scaled to a 70 kg human. “Kupffer” indicate liver Kupffer cells and “Sinusoidal” are liver parenchymal cells. (**A PK model with an additional liver compartment is used for recAP. *T_M_ for recAP corresponds to the liver metabolic time constant.)

Protein	Species	MW	Catabolic organ	T_M_ min	T_P1_ min	T_P2_ min	T_T1_ min	T_T2_ min
Albumin	Human	66,500	Endothelium	16,750	4385	1929	4182	557
Monoclonal Antibody	Human							
Mepolizumab		149,000	Endothelium	21202	8770	3859	4182	557
Infliximab		149,000	Endothelium	11964	8770	3895	4182	557
Alkaline phosphatase	Human							
recAP**		140,000	Liver	1100*	8770	3859	4182	557
Placental		140,000	Liver	7537	8770	3859	4182	557
Amylase	Baboon	57,086	Kidney	100	4385	1929	4182	557
Lipase	Baboon	48000	Kidney	100	4385	1929	4182	557
Alanine Transaminase	Dog	54,600	Sinusoidal	2949	4385	1929	2323	310
Aspartate Aminotransferase	Dog							
Mitochondrial		47,517	Kupffer	49.1	4385	1929	2323	310
Cytoplasmic		46,247	Sinusoidal	689	4385	1929	2323	310
Creatine kinase	Dog	81,000	Kupffer	88.1	6265	2756	2323	310
Lactate Dehydrogenase	Lamb							
LD1 Heart		138,000	Sinusoidal	2871	1370	3859	726	619
LD5 Skeletal muscle		138,000	Kupffer	440	1370	3859	726	619

**Table 2.** Summary of protein pharmacokinetics expressed in terms of the lumped physiological parameters of Figure 1B: plasma metabolic clearance Cl_p_, filtration fractions f_1_, f_2_, and lymph flows L_1_ L_2_. All results are scaled to a 70 kg human using an assumed plasma volume V_p_ = 2800 ml. For each species, the 4 parameters *L*_1_, *L*_2_, *V*_1_ and *V*_2_ are adjusted as described and assumed constant for all proteins studied in that species. Only 2 parameters are adjusted for each protein: Cl_P_ and f ≈f_1_≈f_2_. For human and baboon: V_1_/V_p_ =1.3, V_2_/V_p_= 0.4. For dog and lamb: V_1_/V_p_ =1.0, V_2_/V_p_= 0.3. “Kupffer” indicate liver Kupffer cells and “Sinusoidal” are liver parenchymal cells.(**A PK model with an additional liver compartment is used for recAP. *Cl_p_ for recAP corresponds to the liver metabolic clearance.)

Protein	Species	MW	Catabolic organ	Cl_p_ ml/m	f_1_	f_2_	L_1_ ml/m	L_2_ ml/m
Albumin	Human	66,500	Endothelium	0.167	0.734	0.722	0.87	2.00
Monoclonal Antibody	Human							
Mepolizumab		149,000	Endothelium	0.132	0.367	0.361	0.87	2.00
Infliximab		149,000	Endothelium	0.23	0.367	0.361	0.87	2.00
Alkaline phosphatase	Human							
recAP**		140,000	Liver	66.2*	0.367	0.361	0.87	2.00
Placental		140,000	Liver	0.371	0.367	0.361	0.87	2.00
Amylase	Baboon	57,086	Kidney	27.9	0.734	0.722	0.87	2.00
Lipase	Baboon	48000	Kidney	27.9	0.734	0.722	0.87	2.00
Alanine Transaminase	Dog	54,600	Sinusoidal	0.949	0.530	0.535	1.2	2.71
Aspartate Aminotransferase	Dog							
Mitochondrial		47,517	Kupffer	56.9	0.530	0.535	1.2	2.71
Cytoplasmic		46,247	Sinusoidal	4.06	0.530	0.535	1.2	2,71
Creatine kinase	Dog	81,000	Kupffer	31.7	0.370	0.374	1.2	2.71
Lactate Dehydrogenase	Lamb							
LD1 Heart		138,000	Sinusoidal	0.975	0.53	0.53	3.85	1.35
LD5 Skeletal muscle		138,000	Kupffer	6.35	0.53	0.53	3.85	1.35
